# Telehealth Interventions and Outcomes Across Rural Communities in the United States: Narrative Review

**DOI:** 10.2196/29575

**Published:** 2021-08-26

**Authors:** Michael Butzner, Yendelela Cuffee

**Affiliations:** 1 Department of Public Health Sciences College of Medicine Pennsylvania State University Hershey, PA United States; 2 Program in Epidemiology College of Health Sciences University of Delaware Newark, DE United States

**Keywords:** telehealth, telemedicine, rural health, health outcomes, social determinants of health, eHealth, health care accessibility

## Abstract

**Background:**

In rural communities, there are gaps in describing the design and effectiveness of technology interventions for treating diseases and addressing determinants of health.

**Objective:**

The aim of this study is to evaluate literature on current applications, therapeutic areas, and outcomes of telehealth interventions in rural communities in the United States.

**Methods:**

A narrative review of studies published on PubMed from January 2017 to December 2020 was conducted. Key search terms included telehealth, telemedicine, rural, and outcomes.

**Results:**

Among 15 included studies, 9 studies analyzed telehealth interventions in patients, 3 in health care professionals, and 3 in both patients and health care professionals. The included studies reported positive outcomes and experiences of telehealth use in rural populations including acceptability and increased satisfaction; they also noted that technology is convenient and efficient. Other notable benefits included decreased direct and indirect costs to the patient (travel cost and time) and health care service provider (staffing), lower onsite health care resource utilization, improved physician recruitment and retention, improved access to care, and increased education and training of patients and health care professionals.

**Conclusions:**

Telehealth models were associated with positive outcomes for patients and health care professionals, suggesting these models are feasible and can be effective. Future telehealth interventions and studies examining these programs are warranted, especially in rural communities, and future research should evaluate the impact of increased telehealth use as a result of the COVID-19 pandemic.

## Introduction

Health care is increasingly becoming a technology-driven environment. Telehealth is a remote health care service delivery method and allows for real-time communication between a patient and health care provider [1]. Telehealth is an alternative model of health care service delivery; specifically, it provides opportunities to expand treatment access and reduce barriers to care in underserved and rural areas. Telehealth has been used to promote healthy behaviors and condition management, and there have been promising effects observed, including increasing patient participation and satisfaction and reducing rates of chronic illnesses. In one study, ter Huurne et al [2] conducted a web-based technology study to improve health care service for individuals with chronic eating disorders. In their study, which used a web-based treatment program to improve health care service, 54% of participants completed all program tasks and assignments, and the program significantly improved BMI, body dissatisfaction, quality of life, and physical and mental health [2].

Telehealth is a convenient approach for patients to access health care in the comfort of their own home. Kruse et al [3] conducted a literature review to examine the association of telehealth and patient satisfaction in terms of efficiency and effectiveness. The review concluded that telehealth decreases travel time, improves communication with providers, increases access to care, increases self-awareness, and empowers patients to manage their chronic conditions [3]. From a health care system and provider perspective, benefits include decreased missed appointments, decreased wait times, decreased readmissions, improved medication adherence, and improved quality and timeliness of patient care; in addition, telehealth is a good modality for education [3]. Physician shortage and burnout are common and significant issues in rural areas that can be alleviated by telehealth. Ward et al [4] found that 75% of family physicians in rural areas were covering local emergency departments (ED) as a condition of their practice or hospital privilege. Mandating these conditions discourages physicians from practicing in rural areas; however, the study concluded that telemedicine may help improve the chronic rural workforce shortage by improving physician recruitment and retention [4].

Residents of rural communities across the United States include some of the most vulnerable populations, including individuals with low socioeconomic status, Indigenous communities, children and older adults, and individuals with disabilities [1]. People living in rural communities have limited access to health care, travel long distances to receive care, and/or delay care until after they have a health emergency. Limited access to health care can result in poor health outcomes and is a social and economic burden for both the patient and the health care system. The cost associated with traveling for medical care places an additional burden on the patient, including incurring additional costs for traveling to visits, lost work hours, lower productivity, and increased costs associated with caregiver or childcare support [1]. Telehealth extends the reach of health services and provides the opportunity to reduce barriers to care in rural communities.

The literature assessing the effectiveness of telehealth practices remains ambiguous and many technology interventions are unverified. In rural communities, there are gaps in describing the design and effectiveness of technology interventions and best practices for preventing and treating specific diseases and addressing determinants of health. Synthesizing the current scope and application of telehealth is critical for guiding future interventions and policies. For this article, we defined telehealth as both telehealth and telemedicine and will include mobile health and digital health solutions: electronic health, telemedicine, artificial intelligence, electronic medical record/portal technology, videoconferencing, wearables and biosensors, and remote monitoring tools. The objective of this study was to review and evaluate literature published on the current applications, therapeutic areas, and outcomes of telehealth interventions in rural communities in the United States.

## Methods

For this narrative review, we searched PubMed MEDLINE from January 2017 to December 2020. Key search terms included (“telehealth” AND “telemedicine”), “rural” AND “outcomes.” Filters were applied to identify free full-text studies published in English over a 4-year period to include the most recent evidence available in this research area. We included randomized controlled trials, mixed methods studies, qualitative studies, post hoc analyses, and prospective and retrospective cohort studies. Included studies contained at least one type of telehealth service and one primary measurable outcome and were assessed in rural communities/settings. We excluded systematic reviews, meta-analyses, editorials, conferences, unpublished studies and abstracts, studies outside the United States, and articles that were missing criteria based on the Critical Appraisal Skills Programme criteria. The Critical Appraisal Skills Programme has developed a set of 8 critical appraisal tools to assess the quality of evidence-based research; they have been widely used in previous studies [5].

**Figure 1:**
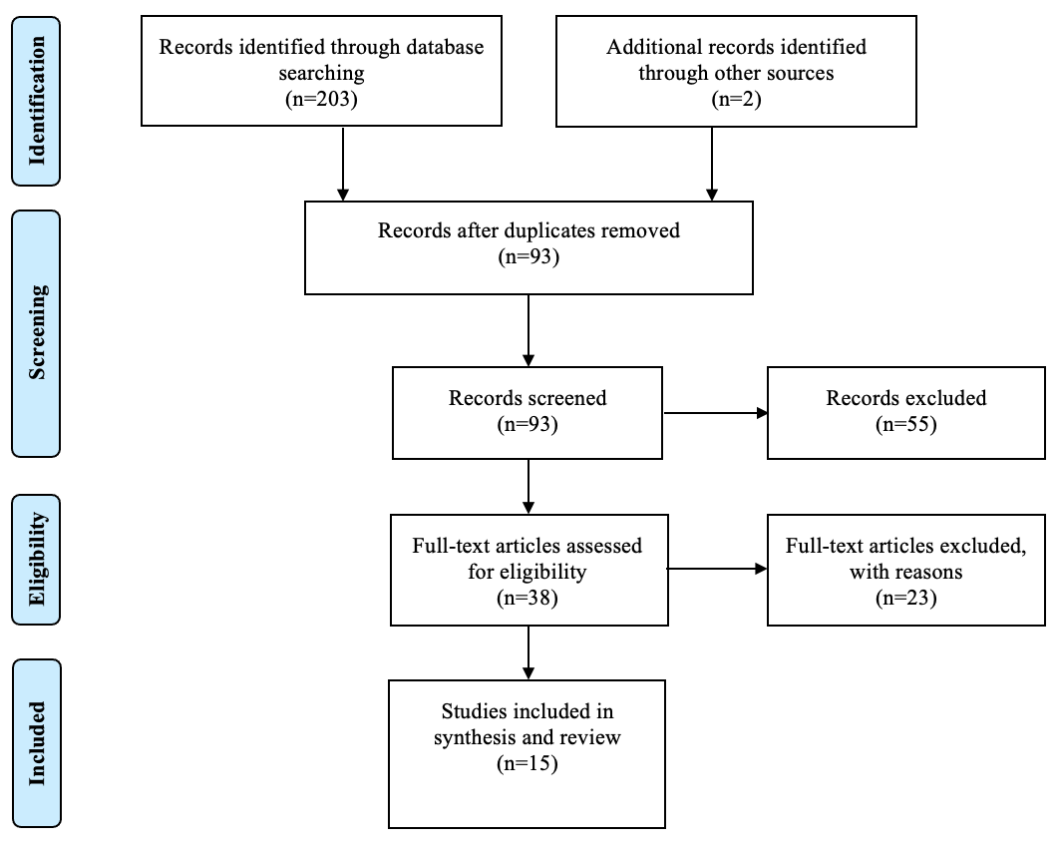
PRISMA flow diagram. PRISMA: Preferred Reporting Items for Systematic Reviews and Meta-Analyses.

To organize our review of the literature, we used Covidence, a review management tool, to conduct a review of titles and abstracts, and a full review of the articles. We used Covidence to exclude duplicate records. Both authors screened studies for relevance based on titles and abstracts. Both authors reviewed the full-text articles of relevant articles for study inclusion. Article discrepancies on study inclusion were resolved through formal discussion and consensus between the two authors. The Critical Appraisal Skills Programme checklists [6] were used to assess the quality and content of the articles. Additionally, we examined the reference lists of all included articles for other relevant references. Figure 1 outlines the article review process. We excluded articles due to irrelevance (n=13), wrong study design (n=8), wrong intervention (n=1), and wrong outcomes (n=1). We extracted the following information from each article: study design, telehealth type, therapeutic area, population, risk of bias, key message, and the primary outcome. We grouped the articles according to study population into the following categories: health care professionals, patients, and health care professionals and patients. This narrative literature review was exempt from ethics review as no human participant protection was required because no human participants were involved in this research.

## Results

### Overview

A total of 38 eligible articles were identified, of which 15 reported telehealth intervention outcomes and were included. The therapeutic areas examined included mental health (n=3), HIV (n=2), reproductive care/women’s health (n=3), osteoporosis (n=1), orthopedics (n=1), acute ischemic stroke (n=1), cancer (n=1), substance use disorder (n=1), ophthalmology (n=1), and emergency medicine (n=1). The majority of the studies (n=9) analyzed telehealth interventions in patients, followed by health care professionals (n=3) and both patients and health care professionals (n=3). Of the patient-centered studies, two studies were specific to veterans, two to Medicare beneficiaries, and one to Medicaid beneficiaries. Table 1 outlines the characteristics the studies included in this review. The outcomes of these studies were focused on the following main themes: feasibility and acceptability of telehealth, diagnostic and treatment validation of telehealth, patient satisfaction and self-confidence, education and training, and telehealth design features including prevalence and access, type of service, and therapeutic area.

**Table 1:** Characteristics of the studies included in the review.

Study	Study design	Telehealth type	Therapeutic area	Outcomes	Risk of bias
Ward, 2018 [4]	Mixed methods	Tele-ED (emergency department)/telemedicine	Emergency medicine	Feasibility and acceptability	Medium
Hicken, 2017 [7]	Randomized	Internet and telephone-based care	Mental health	Feasibility and acceptability	Low
Stringer, 2018 [8]	Mixed methods	Electronic adherence monitor	HIV	Feasibility and acceptability	Low
Uscher-Pines, 2019 [9]	Randomized	Telelactation/telehealth	Reproductive care/women’s health	Feasibility and acceptability	None
Huskamp, 2018 [10]	Retrospective	Tele–substance use disorder/telemedicine	Substance use disorder	Telehealth use	None
Mehrotra, 2017 [11]	Retrospective	Telemental/telemedicine	Mental health	Telehealth use	None
Sinha, 2019 [12]	Qualitative	Telemedicine	Orthopedics	Patient satisfaction	Low
Brecthel, 2018 [13]	Retrospective	Telestroke Network	Acute ischemic stroke	Diagnostic validation	Low
Kapinos, 2019 [14]	Post hoc analysis	Telelactation	Reproductive care/women’s health	Design and demand	None
Talbot, 2019 [15]	Retrospective	Telehealth	Mental health	Prevalence, diagnosis, and type of service	Low
Lewiecki, 2017 [16]	Prospective	TeleECHO	Osteoporosis	Acceptability and self-confidence	Medium
Moeckli, 2017 [17]	Mixed methods	Extension for Community Health Outcomes (ECHO)/telemedicine	HIV	Application and acceptability	Low
Gilbertson-White, 2019 [18]	Mixed methods	Oncology Associated Symptoms and Individualized Strategies (OASIS)	Cancer	Patient needs and satisfaction	Low
Liu, 2019 [19]	Qualitative	Teleophthalmology	Ophthalmology	Feasibility and acceptability	None
Demirci, 2018 [20]	Qualitative	Telelactation/telemedicine	Reproductive care/women’s health	Feasibility and acceptability	Low

### Patients

Of the nine studies that analyzed telehealth interventions in patients, three (33%) of them examined the feasibility and acceptability of telehealth. These studies demonstrated that internet, electronic adherence monitors, and telelactation are feasible and accepted in rural, underserved populations and improve access to care (Table 2). The disadvantages were the following: comparative effectiveness outcomes were not different between caregivers receiving technology interventions and those receiving telephone-delivered support, results were not statistically significant in detecting differences in breastfeeding duration and exclusivity, and technological difficulties, such as loss of connectivity [7-9].

**Table 2:** Key messages from included studies.

Study	Key message
Ward, 2018 [4]	Telemedicine led to decreased staffing costs and improved physician recruitment and retention.
Hicken, 2017 [7]	Technology demonstrated feasibility and acceptability for delivering caregiver support to a group of largely older, rural, spousal caregivers of veterans with dementia.
Stringer, 2018 [8]	Electronic adherence monitor is acceptable and feasible in a rural US setting, but technological difficulties were present and may impede effectiveness.
Uscher-Pines, 2019 [9]	Telelactation participants were breastfeeding at higher rates and telelactation can be implemented in a rural underserved population.
Huskamp, 2018 [10]	There were low use rates of tele–substance use disorder (Tele-SUD) overall. Future studies should evaluate the effect of Tele-SUD on access and outcomes.
Mehrotra, 2017 [11]	States with a telemedicine law and a pro–telemental health regulatory environment had significantly higher rates of telemental health use.
Sinha, 2019 [12]	Telemedicine visits decreased indirect and direct costs, reduced travel time, and resulted in similar patient satisfaction.
Brecthel, 2018 [13]	Telestroke provides less restrictive criteria for clinical risk factors associated with the inclusion of hypertensive patients with stroke for thrombolysis.
Kapinos, 2019 [14]	Telelactation showed both demand for and positive experiences with telelactation in an underserved population.
Talbot, 2019 [15]	Rural Medicaid beneficiaries were more likely to use telehealth services than their urban counterparts, but absolute rates of telehealth use were low.
Lewiecki, 2017 [16]	TeleECHO showed substantial improvement of self-confidence in 20 domains of osteoporosis care and can improve osteoporosis care with greater convenience and lower cost than referral to a specialty center.
Moeckli, 2017 [17]	There was limited uptake of HIV Extension for Community Health Outcomes (ECHO) telemedicine in settings where veterans traveled to distant specialty clinics. Other telemedicine models should be considered for HIV care.
Gilbertson-White, 2019 [18]	Oncology Associated Symptoms and Individualized Strategies (OASIS) is easy to use, contains relevant content, and has pleasing graphics. Rural stakeholders perceived OASIS positively.
Liu, 2019 [19]	Patients and primary care providers have limited familiarity with teleophthalmology for diabetic eye screening and primary care providers reported difficulties with use.
Demirci, 2018 [20]	Telelactation was convenient and efficient, was accepted in rural areas lacking breastfeeding support services, increased maternal breastfeeding confidence, and showed several advantages over in-person and telephone-based support. Telelactation appears to be an acceptable delivery model for lactation assistance in rural areas.

A total of two studies examined the outcomes of telehealth use in Medicare patients with substance use disorder (SUD) and mental health disorders, designated as Tele-SUD and telemental, respectively. Huskamp et al [10] concluded that Tele-SUD has low use rates and is primarily used to complement in-person care and is disproportionately used by those with relatively severe SUD. Mehrotra et al [11] concluded that beneficiaries who received a telemental visit were more likely to be younger than 65 years old, be eligible for Medicare because of disability, and live in a relatively poor community; in addition, states with a pro–telemental health regulatory environment had significantly higher rates of telemental health use than those that did not.

The outcomes of the other four patient-centered studies included patient satisfaction, diagnostic validation, design and demand, and prevalence, diagnosis, and type of service. The advantages of telemedicine visits found in these studies included the following: decreased indirect and direct costs, lower travel costs and travel times, similar patient satisfaction compared to onsite visits, and patient satisfaction among telelactation users [12-14]. In addition, telestroke technology provides less restrictive criteria for clinical risk factors associated with the inclusion of hypertensive patients with stroke for thrombolysis [13]. Talbot et al [15] concluded that rural Medicaid beneficiaries were more likely to use telehealth services than their urban counterparts, psychotropic medication management was the most prevalent use of telehealth, the proportion of users who accessed nonbehavioral health services through telehealth was significantly greater as rurality increased, and significantly higher proportions of telehealth users received services to address attention deficit hyperactivity disorder. There were no direct disadvantages reported in these four studies.

### Health Care Professionals

A total of three studies analyzed telehealth interventions in health care professionals and reported outcomes. Lewiecki et al [16] examined the acceptability of TeleECHO and found that self-confidence in 20 domains of osteoporosis care showed substantial improvement. In addition, they determined that TeleECHO can contribute to alleviating the osteoporosis care crisis by leveraging scarce resources; providing motivated practitioners with the skills to provide better skeletal health care, closer to home, with greater convenience; and being lower cost than referral to a specialty center. Additionally, TeleECHO can be applied to any location worldwide with internet access, allowing access in local time zones and a variety of languages [16].

Another study examined the application and acceptability of a telemedicine intervention called HIV ECHO. Moeckli et al [17] showed a limited adoption of ECHO, which was attributed partly to shifting ownership of care from HIV specialists to primary care providers (PCPs) and low HIV prevalence and long treatment cycles that prevented rapid learning loops for PCPs. More specifically, there was limited uptake of HIV ECHO telemedicine programs in settings where veterans historically traveled to distant specialty clinics [17]. The third study evaluated the feasibility and acceptability of a technology intervention for emergency departments, Tele-ED. Ward et al [4] concluded that Tele-ED hospitals tended to have decreased ED staffing costs—while the hospitals not applying this policy showed continually increasing staffing costs over time—and improved physician recruitment and retention. The only disadvantage to the study was limited uptake of Tele-ED (7/19 hospitals, 37%); however, these results conclude that more hospitals will likely use telemedicine to provide physician backup for advanced practice providers staffing the ED [4].

### Patients and Health Care Professionals

A total of three studies analyzed telehealth interventions in patients and health care professionals. Gilbertson-White et al [18] examined patient needs and satisfaction with an electronic health tool, Oncology Associated Symptoms and Individualized Strategies (OASIS), and concluded that the web application is easy to use, contains relevant content, has pleasing graphics, and was perceived positively. There were infrequent users of OASIS in the group; however, both frequent and infrequent internet users positively evaluated the web application [18]. Liu et al [19] tested the feasibility and acceptability of teleophthalmology and concluded that patients and PCPs have limited familiarity with teleophthalmology for diabetic eye screening. A major disadvantage to teleophthalmology was that PCPs reported significant difficulty identifying when patients are due for diabetic eye screening and could not sufficiently initiate referrals [19].

Another study examined the feasibility and acceptability of telelactation. The advantages of telelactation were as follows: convenient and efficient, provided a needed service in rural areas lacking breastfeeding support services, and increased maternal breastfeeding confidence [20]. The barriers to use included maternal reluctance to conduct video calls with an unknown provider, preference for community-based breastfeeding resources, and technical issues, including limited Wi-Fi in rural areas [20].

## Discussion

### Principal Findings

This review of the literature indicates telehealth is used for a variety of disease states and rural populations across the United States. Health technology interventions are a critical component of health care services in rural areas; they decrease staffing costs, travel costs, and travel time, and increase the ability of residents to seek care (including specialty care) that they otherwise would not be able to access in remote locations. This review explored telehealth in a broad sense and included technology models for clinical use, education and training of health care professionals and patients, and preventive and primary care services. All of these included models have shown feasibility and acceptability in rural populations, validating their importance and potential to improve outcomes and access to care.

Overall, the included studies reported positive outcomes and experiences of telehealth use in rural populations, including acceptability and increased satisfaction; in addition, the technology was considered convenient and efficient. Other notable benefits included decreased direct and indirect costs to the patient (travel cost and time) and health care service provider (staffing), lower onsite health care resource utilization, improved physician recruitment and retention, improved access to care, and increased education and training of patients and health care professionals.

Disadvantages of telehealth interventions included having tele-visits with unknown providers and technological issues such as loss of connectivity and limited Wi-Fi access in rural areas. Several studies reported that comparative effectiveness outcomes between telehealth and traditional visits were not statistically significant; however, these studies also noted telehealth technology was well accepted and implemented in rural, underserved populations and described the importance of testing additional technology interventions in these populations to identify which telehealth programs are most effective.

Feasibility and acceptability are the foundation for implementing new health technologies in any environment or setting. This validation shows that rural populations and underserved communities have the capability to implement telehealth, report satisfaction with telehealth interventions, and describe the interventions as both convenient and effective. Even in articles where telehealth programs did not show statistical significance, these programs have demonstrated feasibility and acceptability. Limitations often associated with technologies (eg, lack of Wi-Fi in remote locations, connectivity issues, and inability of persons to use technology successfully) were reported at low rates, much lower than common perceptions about using these technologies in rural communities. This shows these technologies can be implemented successfully and supports the extension of telehealth programs in rural communities throughout the United States.

A key additional finding of this review regards the appropriate types of telehealth programs. Although these technologies have been verified, these studies consistently highlight the need to test various telehealth programs in specific communities to validate which models are most effective; for example, diagnostic telemedicine versus telehealth video calls will have greater benefits in different settings and populations. It is important that we go beyond the scope of feasibility and acceptability to make sure we use telehealth programs to their full benefit and effect. The benefits have been established but the possibility of expansion of telehealth programs does not rely solely on testing just one model for a population but finding which model fits the community the best.

Additionally, while no articles with a primary outcome of telehealth use for COVID-19 met the criteria for inclusion in this review, the landscape of telehealth and digital health care has changed dramatically since the start of the pandemic. The acceptance of telehealth during the pandemic has accelerated to the point that many of the barriers to telehealth use may have disappeared. To our knowledge, no reviews have been published on this topic in PubMed MEDLINE. Future reviews should focus on the changes in and outcomes of telehealth use in rural communities across the United States due to the COVID-19 pandemic.

The administrative regulations behind telehealth programs, especially in areas where reimbursement for telehealth programs is not enforced by the state, should be highly considered. Requiring insurance companies to provide telehealth services to their patients is key to providing access to health care services in rural communities. Rural communities lack the resources, staff, and expertise to be able to positively affect health care outcomes, health care quality, and health equity. This review highlights the need for additional telehealth program studies and research into long-term real-world outcomes. Evidence-based studies have the potential to establish significance and comparative effectiveness against traditional health care (ie, onsite services). Additionally, state, federal, and local policy should be updated to cover the use of these programs and provide grants and funding for researchers to implement and test these programs in rural, underserved communities to improve access and quality of care.

### Limitations

There are several limitations to this narrative review. Publication bias is possible within this study as we leveraged only PubMed MEDLINE and omitted grey literature such as reports, government documents and releases, working papers, white papers, and evaluations. Searching additional databases would potentially provide additional articles to be in included in this review. Additionally, limiting our search time frame to the past four years excludes earlier publications and data on health technology interventions and outcomes; however, the objective of this review was to report the most recent information on telehealth programs as they have advanced and expanded greatly over the past several years. Lastly, this review only included rural communities in the United States and would not be generalizable to non-US territories or domestic, urban communities, and populations affected by COVID-19. This study is generalizable to rural, underserved populations and potentially to the clinical settings and specific therapeutic areas studied in the included articles.

### Conclusion

This review highlights the current scope of using telehealth interventions in rural populations across the United States. Telehealth models were associated with positive outcomes for patients and health care professionals, suggesting these models can be effective for continuing education and training in the workplace. The findings of this review are limited to rural, domestic communities and are concentrated in specific therapeutic areas of disease. The findings support the existing literature on the need to increase and validate telehealth interventions and further update and implement policies to increase access and provide high-quality telehealth programs. Future telehealth interventions and studies examining these programs are warranted, especially in rural communities, and future research should evaluate the impact of increased telehealth use as a result of the COVID-19 pandemic.

## Abbreviations

ECHO: Extension for Community Health Outcomes
ED: emergency department
OASIS: Oncology Associated Symptoms and Individualized Strategies
PCP: primary care provider
SUD: substance use disorder
